# Electron Energy Loss Processes in Methyl Methacrylate: Excitation and Bond Breaking

**DOI:** 10.1021/acs.jpca.2c09077

**Published:** 2023-03-17

**Authors:** Thomas F. M. Luxford, Juraj Fedor, Jaroslav Kočišek

**Affiliations:** J. Heyrovský Institute of Physical Chemistry of CAS, Dolejškova 3, 18223 Prague, Czech Republic

## Abstract

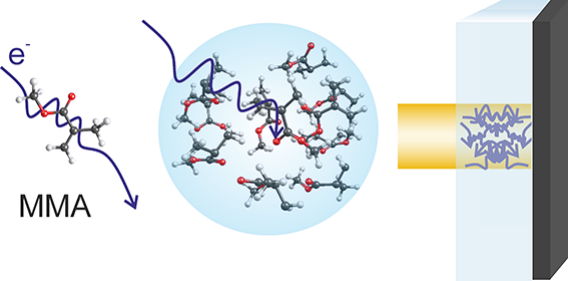

Details of electron-induced chemistry of methyl methacrylate (MMA) upon complexation are revealed by combining gas-phase 2D electron energy loss spectroscopy with electron attachment spectroscopy of isolated MMA and its clusters. We show that even though isolated MMA does not form stable parent anions, it efficiently thermalizes the incident electrons via intramolecular vibrational redistribution, leading to autodetachment of slow electrons. This autodetachment channel is reduced in clusters due to intermolecular energy transfer and stabilization of parent molecular anions. Bond breaking via dissociative electron attachment leads to an extensive range of anion products. The dominant OCH_3_^–^ channel is accessible via core-excited resonances with threshold above 5 eV, despite the estimated thermodynamic threshold below 3 eV. This changes in clusters, where M_*n*_OCH_3_^–^ anions are observed in a lower-lying resonance due to neutral dissociation of the ^1^(n, π*) state and electron self-scavenging. The present findings have implications for electron-induced chemistry in lithography with poly(methyl methacrylate).

## Introduction

Methyl methacrylate (MMA) is a building block of poly(methyl methacrylate) (PMMA), a common photoresist for both electron and photon beam lithography.^1−3^ PMMA can be used as a positive photoresist, when PMMA is damaged by the action of electrons or photons and its removal by solvent is enhanced, or as a negative photoresist, when it is densified by the action of electrons or photons and the desired pattern remains on the substrate upon solvation.^4−8^ The first case usually occurs via polymer chain scission at low exposures, while the second case occurs at high exposures where a high number of secondary reactive species is expected to form and induce more cross-linking in the resist.^9^ Higher exposure can lead to the formation of carbonaceous structures by PMMA “baking”.^10,11^

The primary electrons and photons used in lithography typically produce large amounts of secondary electrons in the resist. These cause *secondary electron blur*, caused by modification (damage/cross-linking) of the resist.^12−14^

The effects of primary and secondary species in bulk are difficult to separate. Ionizing radiation was observed to remove the ester group and further induce its fragmentation.^15,16^ The irradiation of bulk PMMA by low-energy electrons did not result in significant macroscopic changes;^17^ however, decomposition products similar to high-energy irradiation were observed in thermal desorption experiments, such as H_2_, CO, and CO_2_.^18^ Since the lifetime and mean free path of secondary low-energy electrons in PMMA are extremely short,^19,20^ the identification of fundamental mechanisms of PMMA decomposition requires time-resolved radiolysis studies.^21^ These show an indisputable role of transient negative ions in the process induced by interaction with secondary low-energy electrons and decay via release of CH_3_ neutral radicals and polymer scission.^22^ Anions are well-known to activate polymerization reactions^23^ in various precursors, including acrylates.^24−26^ For MMA, anion-induced polymerization was explored in clusters upon electron transfer,^27,28^ showing that the cationic counterion can significantly enhance the process.^28^ It is therefore generally accepted that low-energy electrons do play an important role in the PMMA modification.

The understanding of these elementary electron-induced processes is being exploited for the rational design of chemically amplified resists.^29,30^ Recently, the reactivity of precursor molecules toward high-energy radiation^31^ as well as low-energy electrons^32^ has been suggested as an important parameter for the rational design of lithography precursors.

Somewhat surprisingly, information about the processes induced by low-energy electrons in MMA, the building block of PMMA, is very limited. The only study of the isolated molecule is that of Schafer and co-workers on electron transmission and electron energy loss of MMA.^33^ The data show close to 0 eV electron affinity of MMA and three peaks in the electron energy loss spectrum, corresponding to electronic excitation of the molecule. MMA clusters were studied in collisions with Rydberg-state Kr atoms^27^ (equivalent to near 0 eV electron attachment) and showed pronounced formation of MMA dimers. The observation of dimers was assigned to an exothermic anion-induced polymerization reaction.

Here we report data on low-energy (<12 eV) electron collisions with gas-phase MMA and its clusters. In terms of energy loss processes, we focus on vibrational excitation, which has not been explored previously. In terms of bond-breaking reactions, we focus on the anionic pathways via dissociative electron attachment (DEA). First, DEA is a resonant process mediated by the formation of transient negative ions (resonances) and can thus be extremely efficient. Second, the electron affinity of one of the products allows for a significant reduction of dissociation energy. In many cases, the presence of a slow electron in the vicinity of the molecule can lead to fragmentation, as is known for organometallic precursors used in nanofabrication.^34−37^ Comparing the results for the gas phase and clusters allows us to elucidate the basic effects of the aggregation on the electron-induced chemistry.

## Methods

MMA (liquid at standard conditions) was purchased from Sigma-Aldrich at a stated purity of 99% and was used in all experiments without further purification.

### Isolated Molecule: Electron Energy Loss Spectra

The electron energy loss spectra were recorded on an electrostatic spectrometer.^38,39^ The electrons produced from a heated iridium filament passed through a double-hemispherical electron monochromator to reduce their energy distribution function down to 18 meV as determined from the width of the elastic peak in helium. The electrons accelerated to incident electron energy ϵ_i_ were scattered on an effusive beam of MMA gas, and their residual energy ϵ_out_ was analyzed with a double-hemispherical electron analyzer. The difference between the measured energy of the incoming electron ϵ_i_ and energy of outgoing electron ϵ_out_ is the energy loss Δϵ. All of the current data were recorded at a scattering angle of 135°. The energy of the incident beam was calibrated on the 2^2^S resonance in helium at 19.365 eV.

### Isolated Molecule: Dissociative Electron Attachment

DEA to isolated MMA was recorded on a troichoidal electron monochromator–quadrupole mass spectrometer (TEM-QMS) apparatus.^40,41^ MMA was held in a glass container at room temperature. Vapor from the sample passed through a Swagelok inlet line and then through a 1 cm long, 0.3 mm diameter capillary directly to the region of interaction with electrons. A needle valve was used to regulate the pressure of gas-phase MMA and keep a constant pressure of 1 × 10^–3^ mbar before the capillary, resulting in a reaction chamber pressure of 2 × 10^–6^ mbar. A continuous beam of electrons was produced using an yttria-coated iridium stripe cathode. The electrons were energy-selected using a troichoidal electron monochromator and focused to the interaction region using a set of electrostatic lenses. Anions formed in the interaction region were directed toward a quadrupole mass filter, which selected ions at a single *m*/*z* to be counted using a channeltron electron multiplier detector. Ion yield curves were obtained by measuring the ion yield as a function of electron energy at a fixed *m*/*z*.

### Clusters: Dissociative Electron Attachment

DEA to clusters of MMA was recorded using the cluster beam (CLUB) apparatus.^42,43^ MMA was kept in a glass cell inside a metal cylinder kept at a temperature of 45 °C in front of a 90 μm nozzle heated to 60 °C. After expansion into vacuum (the pressure during the experiment was 1 × 10^–5^ mbar), a skimmed molecular beam interacted with electrons in a TOF chamber (at a pressure of 1 × 10^–9^ mbar). The electrons were produced from a simple electron gun and collimated by permanent magnets to allow interaction at low energies. The electron current in experiments under 1.5 eV is low, and the electron energy distribution function has a typical fwhm around 0.7 eV.^44^ Therefore, the measured electron-energy-dependent ion yields have rather qualitative than spectroscopic character. Product anions were extracted from the interaction region into a reflectron time-of-flight mass spectrometer. Mass spectra were recorded for electron energies of 0–15 eV in steps of 0.25 eV.

### Theoretical Methods

Calculations of the threshold energies for different DEA reaction pathways were performed at the B3LYP/aug-cc-PVTZ level of theory using the Gaussian 16 software.^45^ Geometry optimizations and single-point energy calculations were performed for the MMA molecule and possible anion and neutral dissociation products. From the single-point energies, the energetic threshold, *E*_th_, for a reaction pathway was obtained using the formula
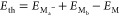
1where *E*_M_a_^–^_ and *E*_M_b__ are the sums of electronic and zero-point energies of the anion and neutral fragments, respectively (if there are multiple neutral fragments, then *E*_M_b__ is the sum of the electronic and zero-point energies for all neutral fragments), and *E*_M_ is the energy of the parent MMA molecule.

**Figure 1:**
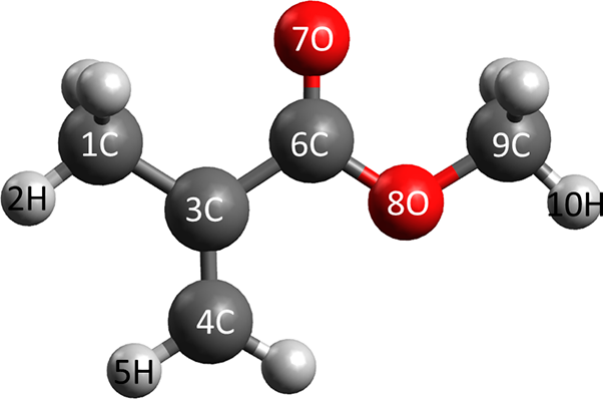
Structure of the lowest-energy conformer of MMA.

Figure 1 shows the lowest-energy conformer of MMA.^46^ While it is expected that multiple conformers of the molecule are present under the experimental conditions,^46^ for simplicity all calculations were based on this structure.

## Results and Discussion

### Electron Energy Loss in the Gas Phase

**Figure 2:**
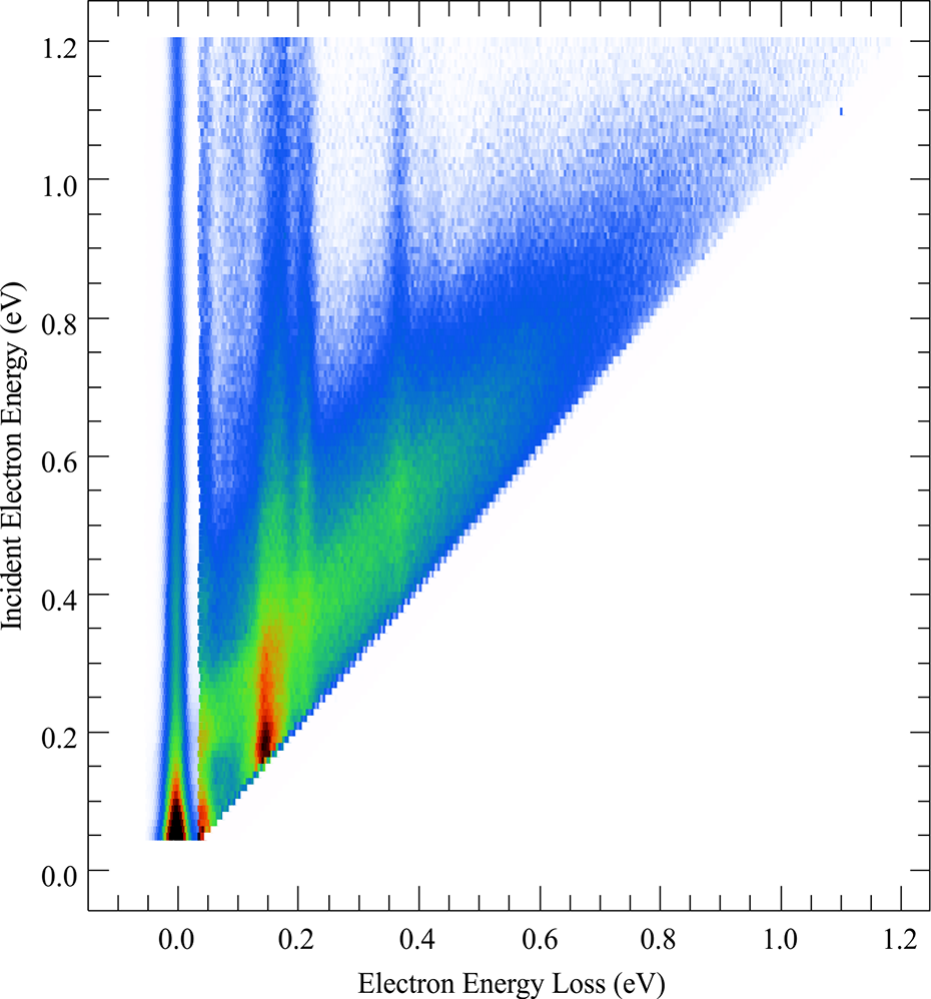
Two-dimensional electron energy loss spectrum for methyl methacrylate.

Figure 2 shows the two-dimensional electron energy loss spectrum (EELS). It is constructed from individual energy loss spectra recorded at increasing incident electron energies with 10 meV increments. This color-coded map provides detailed information about the target states and the internal energy flow upon electron scattering.^34,47−50^ The *y* axis shows the incident energy, ϵ_i_, and the *x* axis shows the energy loss, Δϵ.

The most prominent feature in the 2D plot is the broad diagonal strip running along the bottom of the plot. These are electrons that have a constant (low) ϵ_out_ over a broad range of incident energies. This shows a strong propensity for MMA to thermalize electrons—an electron with ϵ_i_ is temporarily attached, its energy is randomized over the vibrational degrees of freedom, and it is emitted (thermally) with low ϵ_out_. This emission is not necessarily purely thermal: mode-specific vibrational autodetachment can be also active,^48,51^ where the outgoing thermal electron selectively de-excites a specific vibrational mode. The fact that the spectrum of emitted electrons does not peak at ϵ_out_ = 0 eV (the diagonal strip is rather broad with the maximum around ϵ_out_ ≈ 0.2 eV) suggests that this might be indeed the case. However, the distinction between the purely thermal detachment and the mode-specific autodetachment is complicated by many vibrational degrees of freedom of MMA and by the fact that the sensitivity of the hemispherical analyzer is difficult to control at very low ϵ_out_ (typically, the sensitivity steeply decreases for electrons with ϵ_out_ less than 50 meV).

**Figure 3:**
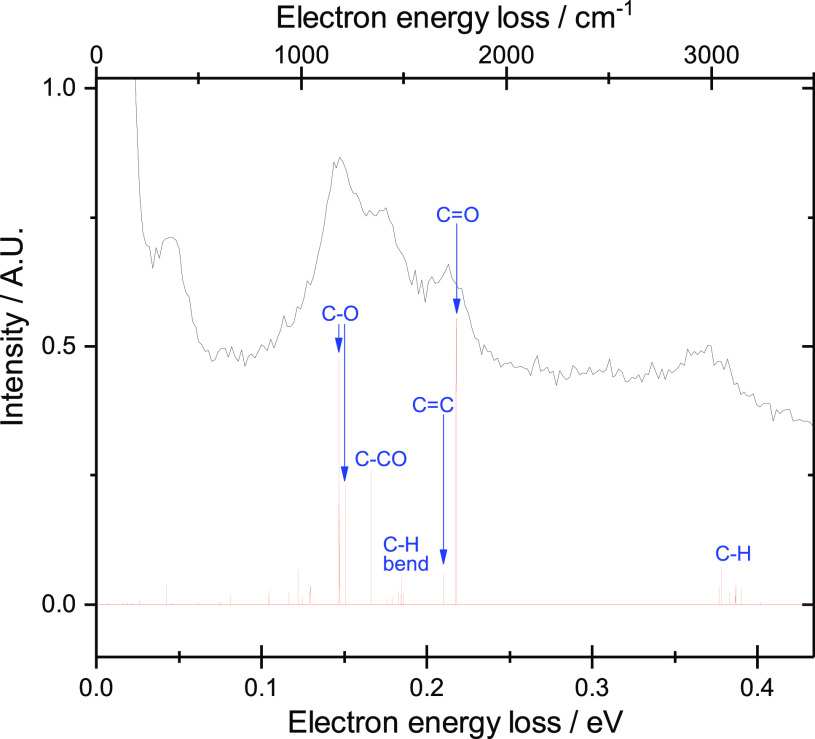
Black: electron energy loss spectrum for MMA recorded at constant ϵ_out_ = 0.2 eV. Red: IR spectrum for MMA simulated using Gaussian 16 at the B3LYP/aug-cc-pvtz level of theory.

A second feature in the 2D plot in Figure 2 is the vertical strips (showing constant energy losses), which correspond to direct electron-impact excitation of vibrational levels. The vertical strip at zero energy loss corresponds to elastic scattering, while the vertical strips at higher energy losses correspond to vibrationally excited states of MMA. To ease the level assignment, Figure 3 shows a separately recorded 1D EELS, measured at a constant outgoing energy of 0.2 eV, which corresponds to an intensity profile of the 2D spectrum along the diagonal ϵ_out_ = 0.2 eV. It reveals vibrations that are excited close to their threshold, as soon as the electron has enough energy to access them. It is well-established that the cross section for such excitation reflects the IR activity of the normal modes,^52^ and it is therefore useful to compare this spectrum to an IR spectrum for MMA. Figure 3 shows the diagonal intensity profile plotted together with an IR spectrum simulated at the B3LYP/aug-cc-pvtz level of theory, which is used to assign the different peaks. The strongest vibrational modes that are excited upon electron impact are C–O, C=O, and C–CO stretching groups of vibrations. Also visible are the C–H stretch modes. Bending is excited only weakly. This simulated spectrum closely matches the experimental IR spectrum for MMA measured by Sugumaran and Karim.^53^ The simulated IR spectrum and the electron energy loss spectrum (note that the “background” in the EELS spectrum corresponds to the thermal electrons discussed above) have clear similarities, with peaks at the same energies.

**Figure 4:**
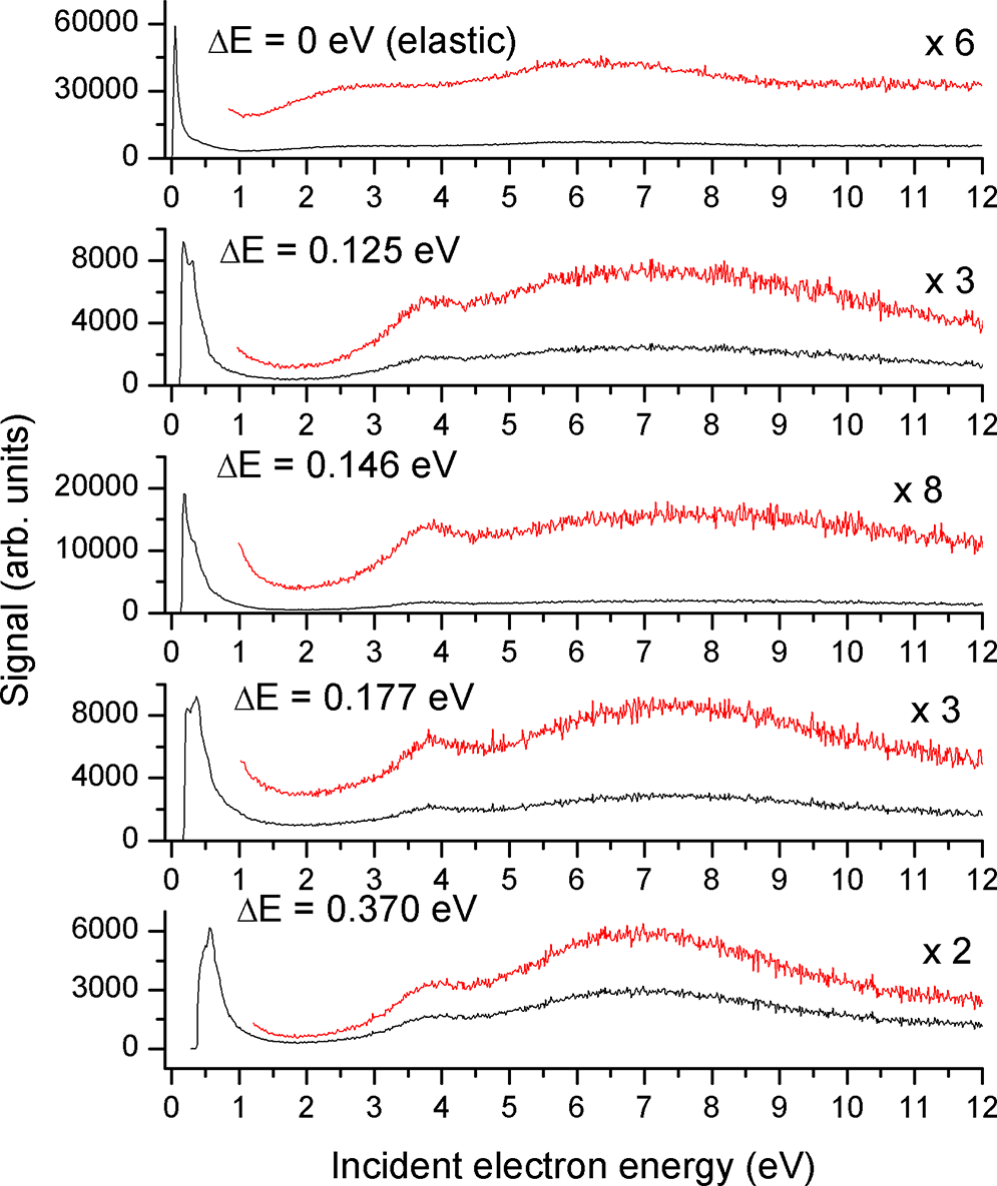
Excitation curves for different constant energy losses as functions of incident electron energy.

The spectra in Figure 4 show the excitation curves of the four strongest vibrations, showing the probability that the vibration is excited as a function of the incident energy. They correspond to vertical intensity profiles of the 2D spectrum but were separately recorded in a longer ϵ_i_ range. The character of all excitation curves is similar. As discussed above, there are intense peaks close to threshold for all vibrations. Apparent structures of some of the threshold peaks (e.g., Δ*E* = 125 and 146 meV) are artifacts of the finite energy resolution and of the closely spaced vibrational levels of MMA (if a neighboring, not fully resolved vibration has a threshold peak, it is manifested as a shoulder on the main peak). Two structures are observed at higher energies of the incident electron, ϵ_i_, a narrower one around 3.8 eV and a very broad one (width of several eV) with center around 7 eV. These are due to the formation of shape resonances.

**Figure 5:**
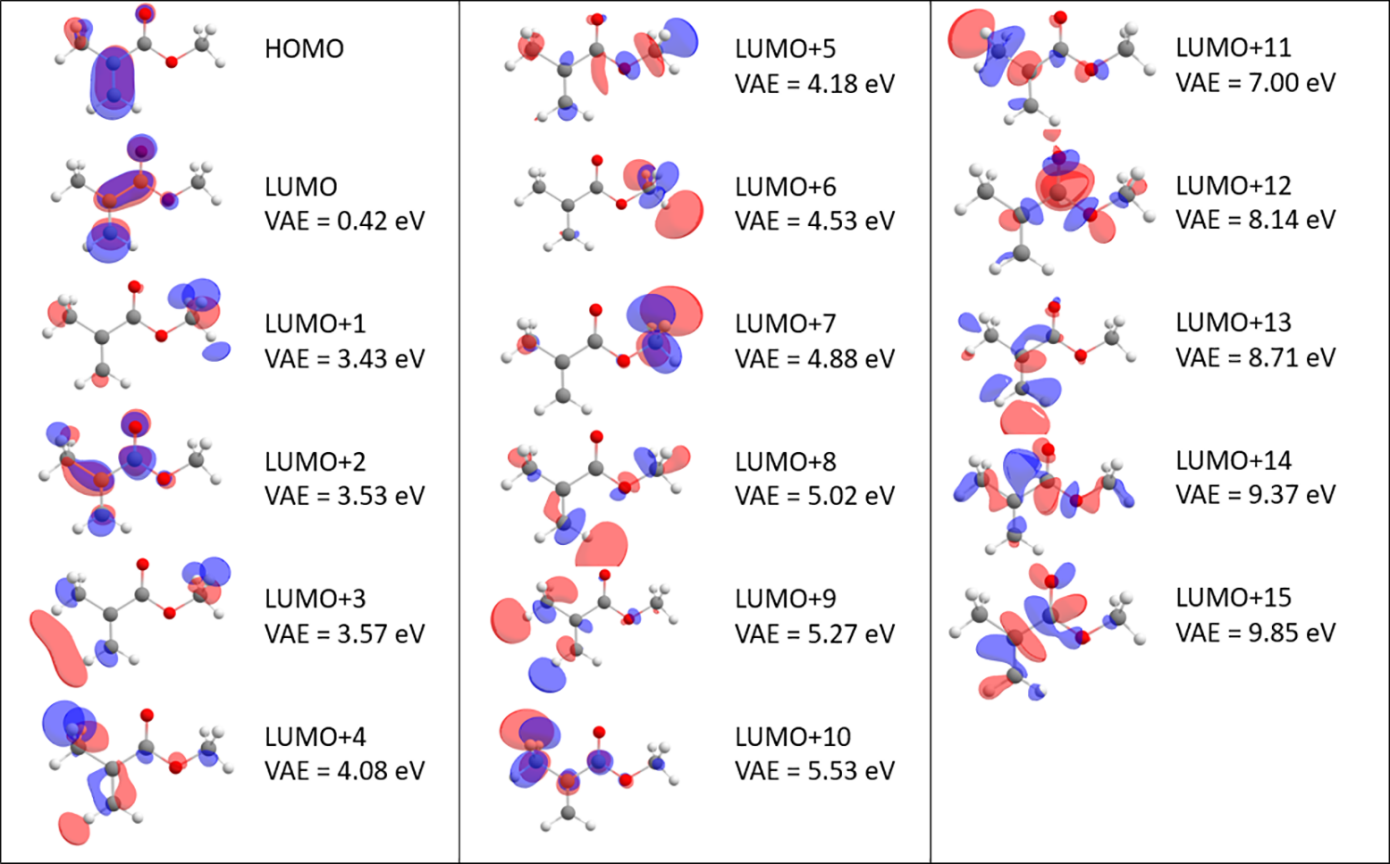
Virtual orbitals and associated vertical attachment energies. Molecular structures were calculated at the B3LY/aug-cc-pvtz level, and orbital energies and structures were calculated at the HF/6-31G level. Vertical attachment energies were obtained from Gallup and Chen scaling of the orbital energies.

Resonances are states embedded in a continuum, and their proper theoretical characterization requires either scattering calculations^50^ or extensions of quantum chemistry methods.^54^ To assign the peaks in Figure 4, we used the empirical scaling method of Gallup and Chen,^55^ which approximately relates energies of virtual orbitals with the expected positions of corresponding resonances (vertical attachment energies (VAEs)). Figure 5 shows VAEs of MMA obtained by such scaling. For the two π* virtual orbitals (LUMO and LUMO+2), the scaling yields VAEs of 0.42 and 3.53 eV. The higher one can correspond to the 3.8 eV peak in the excitation curves. We thus assign that feature as arising from the LUMO+2 π* resonance. Both LUMO+3 and LUMO+4 have σ* character and are thus expected to be much broader than the observed resonance (due the very low barrier toward electron autodetachment). There is no structure either in the excitation curves or in the 2D spectrum which would correspond to the presence of a low-lying π* resonance corresponding to temporal population of the LUMO. Note that the threshold peaks are due to direct dipole excitation and the seeming structure on them is the artifact of the finite beam resolution. The π* shape resonance corresponding to the LUMO could in principle overlap with the threshold peaks, However, in such case a regular structure would be expected on the threshold peak.^56^ We find it more plausible that the scaling places the LUMO state to the continuum erroneously and that this anion state is slightly bound. It may play a role in the thermal electron emission (diagonal feature in the 2D spectrum). This is in agreement with the finding of Schafer et al.^33^ about near-zero electron affinity of MMA. Finally, the very broad band in the excitation curves around 7 eV corresponds to several overlapping σ* CC and CH resonances (presumably with large electronic widths).

### DEA in the Isolated Molecule

**Figure 6:**
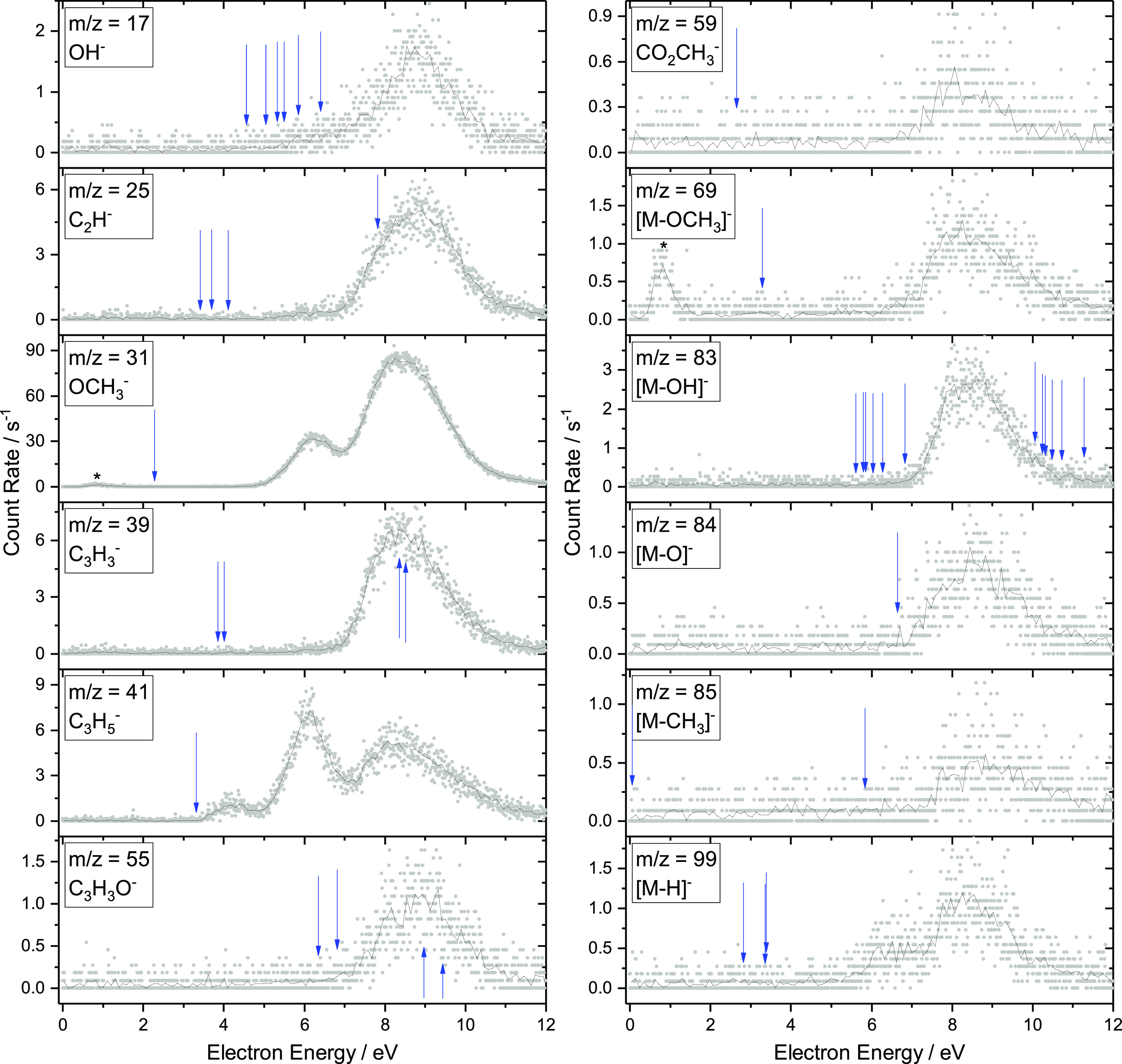
Energy-dependent anion yield curves for electron attachment to MMA obtained using the TEM-QMS apparatus. Solid lines represent 10-point averages of the experimental data. Peaks marked with * are confirmed to arise due to background processes. Blue arrows show the threshold energies in Table 1.

Using the TEM-QMS experiment, a total of 12 fragment anions were observed upon DEA, showing a rich chemistry that is induced by electron attachment. Of the products, OCH_3_^–^ is the most intense. The ion yield curves are shown in Figure 6. For all observed products, there is a peak in the ion yield curves at an incident electron energy of 8.5 eV. Furthermore, some species have additional peaks at 4 and 6 eV. For each observed anion, a number of potential dissociation channels were identified, and for each, the thermodynamic threshold was calculated to help understand the experimental results. These thresholds are shown in Table 1 and as blue arrows on the spectra in Figure 6.

**Table 1:** Potential Fragmentation Pathways of MMA and the Threshold Energies Calculated at the B3LYP/aug-cc-pVTZ Level of Theory

anion	neutral products^a^	bonds broken^b^	*E*_th_/eV
OH^–^	[M – OH] (d)	1C–2H, 6C–7O	4.56
OH^–^	[M – OH] (d)	6C–7O, 9C–10H	5.04
OH^–^	[M – OH] (q)	1C–2H, 6C–7O	5.32
OH^–^	[M – OH] (d)	4C–5H, 6C–7O	5.50
OH^–^	[M – OH] (q)	6C–7O, 9C–10H	5.85
OH^–^	[M – OH] (q)	4C–5H, 6C–7O	6.40
C_2_H^–^	CO_2_CH_3_, CH_4_	1C–3C, 3C–6C, 4C–5H	3.42
C_2_H^–^	HCO_2_CH_3_, CH_3_	1C–3C, 3C–6C, 4C–5H	3.69
C_2_H^–^	CH_3_CO_2_CH_3_, H	1C–3C, 3C–6C, 4C–5H	4.11
C_2_H^–^	CO_2_CH_3_, CH_3_, H	1C–3C, 3C–6C, 4C–5H	7.82
OCH_3_^–^	[M – OCH_3_]	6C–8O	2.28
C_3_H_3_^–^	CO_2_CH_3_, H_2_	1C–2H, 3C–6C, 4C–5H	3.86
C_3_H_3_^–^	CO_2_CH_3_, H_2_	3C–6C, 4C–5H, 4C–5H	4.01
C_3_H_3_^–^	CO_2_CH_3_, 2H	1C–2H, 3C–6C, 4C–5H	8.36
C_3_H_3_^–^	CO_2_CH_3_, 2H	3C–6C, 4C–5H, 4C–5H	8.51
C_3_H_5_^–^	CO_2_CH_3_	3C–6C	3.31
C_3_H_3_O^–^ (s)	OCH_3_, CH_2_ (t)	3C–4C, 6C–8O	6.34
C_3_H_3_O^–^ (s)	OCH_3_, CH_2_ (s)	3C–4C, 6C–8O	6.81
C_3_H_3_O^–^ (t)	OCH_3_, CH_2_ (t)	3C–4C, 6C–8O	8.96
C_3_H_3_O^–^ (t)	OCH_3_, CH_2_ (s)	3C–4C, 6C–8O	9.44
CO_2_CH_3_^–^	C_3_H_5_	3C–6C	2.64
[M – OCH_3_]^−^	OCH_3_	6C–8O	3.29
[M – OH]^−^ (s)	OH	1C–3H, 6C–7O	5.61
[M – OH]^−^ (s)	OH	6C–7O, 9C–10H	5.79
[M – OH]^−^ (t)	OH	1C–3H, 6C–7O	5.85
[M – OH]^−^ (t)	OH	6C–7O, 9C–10H	6.04
[M – OH]^−^ (s)	OH	4C–5H, 6C–7O	6.27
[M – OH]^−^ (t)	OH	4C–5H, 6C–7O	6.83
[M – OH]^−^ (s)	O, H	1C–3H, 6C–7O	10.06
[M – OH]^−^ (s)	O, H	6C–7O, 9C–10H	10.24
[M – OH]^−^ (t)	O, H	1C–3H, 6C–7O	10.31
[M – OH]^−^ (t)	O, H	6C–7O, 9C–10H	10.49
[M – OH]^−^ (s)	O, H	4C–5H, 6C–7O	10.73
[M – OH]^−^ (t)	O, H	4C–5H, 6C–7O	11.28
[M – O]^−^	O	6C–7C	6.65
[M – CH_3_]^−^	CH_3_	8O–9C	0.05
[M – CH_3_]^−^	CH_3_	1C–3C	2.21
[M – H]^−^	H	1C–2H	2.81
[M – H]^−^	H	9C–10H	3.36
[M – H]^−^	H	4C–5H	3.39
M^–^			–0.07

a (s), (d), (t), and (q) designate singlet, doublet, triplet, and quartet states, respectively.

b The bonds broken are between the atoms as labeled in Figure 1.

For the products OCH_3_^–^, C_3_H_5_^–^, CO_2_CH_3_^–^, [M – OCH_3_]^−^, and [M – O]^−^, the simplest fragmentation mechanism involves the cleavage of one covalent bond. In each of these cases, the threshold energy calculations show that this mechanism is thermodynamically accessible, so it is the most likely reaction to occur.

For [M – H]^−^, there are three possible sites from which the H atom may dissociate, all of which are thermodynamically accessible and thus may contribute to the signal. Similarly, for [M – CH_3_]^−^ there are two energetically accessible sites for loss of CH_3_.

C_3_H_3_O^–^ may be formed by the cleavage of two covalent bonds, to be formed together with neutral OCH_3_ and CH_2_ cofragments. Both the CH_2_ neutral and C_3_H_3_O^–^ anion contain a carbene group and thus may exist in either a singlet or triplet electronic state. This means that there are four possible combinations of products. The calculated energy thresholds for singlet C_3_H_3_O^–^ are lower than the observed ones, regardless of the multiplicity of the CH_2_. On the other hand, the threshold energy for triplet C_3_H_3_O^–^ is higher than observed, meaning that it is energetically inaccessible, so only the singlet will be present.

For OH^–^, there are three possible locations from which the H atom can be abstracted. The resulting [M – OH] neutral cofragment contains an unpaired electron and a carbene group and thus can either be present in a doublet or quartet state. This gives a total of six possible reaction channels, all of which are thermodynamically accessible. The [M – OH]^−^ anion may be formed through the same six channels as OH^–^, but with the charge remaining on the [M – OH] body (with singlet and triplet states, rather than doublet and quartet states) and a neutral OH radical. All six of these channels are thermodynamically accessible. Alternately, the O and H atoms may dissociate separately, but these channels have a much higher thermodynamic threshold and are therefore energetically inaccessible.

C_3_H_3_^–^ can be formed by the dissociation of neutral CO_2_CH_3_ and two additional H atoms. There are two possible final structures of C_3_H_3_^–^, which are both energetically accessible when formed in combination with a single H_2_ molecule. C_3_H_3_^–^ release together with two H atoms is only possible at higher energies corresponding to the tail of the experimentally observed peak. Similarly, C_2_H^–^ may be formed by the dissociation of neutral CO_2_CH_3_, CH_3_, and H. With three separate neutral cofragments, the threshold energy is above the lowest electron energy at which signal is observed. On the other hand, when any two of CO_2_CH_3_, CH_3_, and H combine, the threshold energy decreases considerably, and the process becomes energetically accessible.

The energetic thresholds determine only the lowest energies at which given ions can be observed. The actual peak positions in Figure 6 are determined by the formation of resonances. The 3.8 eV π* shape resonance (temporal occupation of LUMO+2) identified in the previous section gives rise to only the C_3_H_5_^–^ fragment. The σ* “mountain” visible in Figure 4 is not expected to give rise to a detectable DEA signal because these shape resonances will have prohibitively large electronic width (electrons will autodetach prior to the dissociation). Instead, core-excited resonances will play a role in the energy range above 5 eV. Schafer et al.^33^ identified the ^1^(ππ*) state of neutral MMA at 6.3 eV. This state can serve as a parent state of the DEA band around 6 eV yielding OCH_3_^–^, C_3_H_5_^–^, and [M – H]^−^. The dominant DEA band at 8.5 eV has to originate from a core-excited resonance, but we are not able to determine its configuration. A rich fragmentation pattern of such resonances is indeed expected.^57,58^

The parent ion was not observed in the gas-phase experiment. This is not unusual, as DEA and autodetachment typically occur on a shorter time scale than the detection. The electron affinity of MMA was calculated here to be 0.07 eV (B3LYP/aug-cc-PVTZ). This means that electron attachment to form the parent ion is almost isoenergetic. The presently calculated MMA dipole moment of ∼1.8 D (B3LYP/aug-cc-PVTZ) can theoretically result in attachment of electrons via a dipole-supported state,^59^ but the practical observation limit is usually set in the 2–2.5 D range.^60−62^ We can see that both the electron affinity and dipole moment are close to the limit, and unambiguous assignment of the nature of the isolated MMA anions is therefore not possible using the present experimental approaches or computational methods. We can only conclude that the lifetime of the anions will be much shorter than detection times in our QMS setup (100 ms) and much longer than typical vibrational periods of the molecule (ps), as indicated by thermal electron emission upon electron attachment in 0–0.6 eV range observed using EELS in Figure 2.

### DEA in Clusters

**Figure 7:**
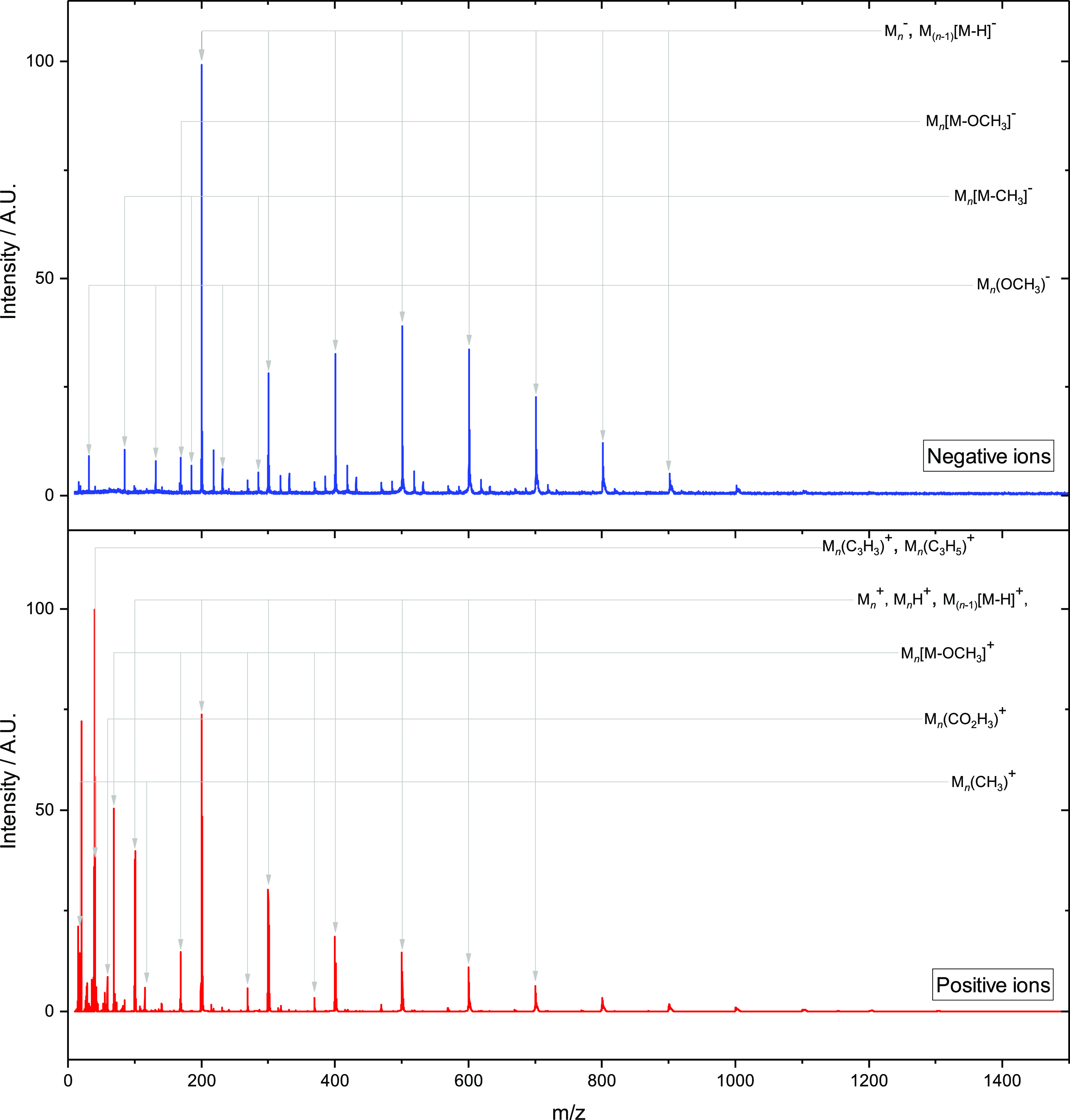
Mass spectra for MMA clusters recorded using the CLUB apparatus. Top: negative ion mass spectrum compiled from individual mass spectra recorded at electron energies of 0–15 eV. Bottom: positive ion mass spectrum recorded at an electron energy of 70 eV. (Detailed sections of the spectra in the *m*/*z* 10–810 range are given in the Supporting Information.)

**Figure 8:**
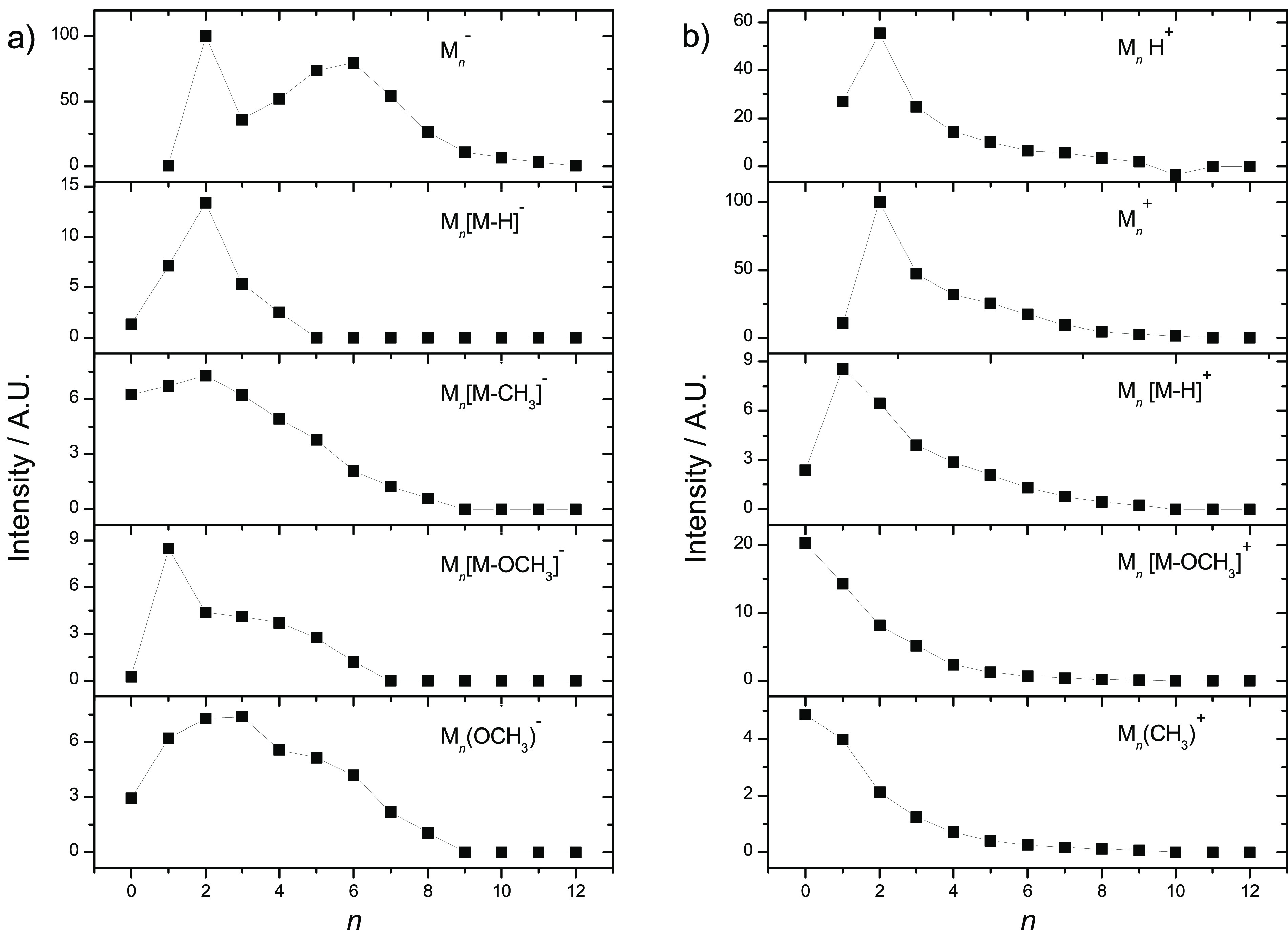
Cluster size distributions of different anion products from EA to MMA clusters. Intensities were obtained by fitting Gaussian functions to the peaks in the mass spectrum. The intensities are scaled to the most intense species. Where there is no visible peak in the mass spectrum, the species is assigned an intensity of 0.

DEA to clusters of MMA molecules was investigated using the CLUB apparatus. The negative ion mass spectrum is shown in the upper panel of Figure 7. The cluster size distributions for intense fragment anion species (>5% of maximum in MS), obtained as integrals of the Gaussian functions fitted to individual peaks in Origin, are shown in Figure 8.

Comparing the DEA of the isolated molecule to that of clustered MMA, we can see several anions produced via the same dissociative mechanism, such as M_*n*_(OCH_3_)^−^, M_*n*_[M – OCH_3_]^−^, M_*n*_[M – CH_3_]^−^, M_*n*_[M – H]^−^, and C_3_H_5_^–^.

On the other hand, the ions C_2_H^–^, C_3_H_3_^–^, C_3_H_3_O^–^, CO_2_CH_3_^–^, [M – OH]^−^, and [M – O]^−^ were observed in the measurement of the isolated molecule, but the corresponding complexes with M_*n*_ are not present in the measurement of the clusters. Formation of these ions may be suppressed by the cluster environment, or these ions may still be present but are simply below the detection threshold of the instrument. We can see from the gas-phase measurements that the intensities of these anions are below 1% of the OCH_3_^–^ signal, which roughly corresponds to the background level in the cluster measurement. OH^–^ is visible in the cluster measurement, but this appears to be mainly signal from residual water in the experiment rather than a DEA product of MMA.

By far the most intense progression in the mass spectrum of cluster anions corresponds to the parent ion, M_*n*_^–^. The intact molecular anion M^–^ was not observed for DEA to the isolated molecule. The presence of several MMA units clearly leads to stabilization of the excess electron. Instead of thermal electron ejection, which is observed in the gas phase, the energy is dissipated into the cluster. This phenomenon is commonly observed for clusters^63,64^ and may be associated with evaporation of neutral monomer units.^65^ We can also see a low-intensity progression of M_*n*_(H_2_O)^−^ anions, likely formed by clustering of MMA molecules with water that is present as an impurity in the sample.

It is interesting to explore the abundances of M_*n*_X^–^ ions as a function of *n*, as shown in Figure 8. First, we can see a strong magic peak at *n* = 2 for M_*n*_^–^ parent anions. Similar behavior was observed also by Tsukuda^27^ and assigned to anion-induced polymerization of MMA. In the present experiment we can compare the negative ion spectrum to that of positive ions measured at an electron energy of 70 eV for the same cluster beam conditions. The positive ion mass spectrum is shown in the bottom panel of Figure 7, and the corresponding dependences on cluster size are shown in Figure 8. The parent cation M_*n*_^+^ exhibits similar enhancement of the dimer ion signal, indicating that the enhancement is caused by a preference for dimerization already for neutral MMA and higher abundance of the dimers in the molecular beam. At the same time, progressions of M_*n*_[M – H]^−^ (Figure 8a) and M_*n*_H^+^ (Figure 8b) with highest intensity at *n* = 2 indicate that these particular dimer structures are more stable in the ionic state and may be formed by decay of various *n* > 2 clusters.

A similar dependence of the cluster size to that of parent anions is observed also for M_*n*_[M – OCH_3_]^−^, which indicates that these ions are formed by simple ejection of OCH_3_ from the cluster. For positive ions, this fragment shows a smooth decreasing dependence, indicating an exothermic process that does not conserve the dimer preference of the neutral precursor. For the rest of the intense ion progressions, the dimer enhancement is suppressed. The mixed clusters of MX^–^ and M_2_X^–^ for X = [M – H], OCH_3_, and [M – CH_3_] are of similar intensity. While the decrease of intensities for X = OCH_3_ and [M – CH_3_]-type anions is rather slow, it is fast for [M – H] ions. Most probably this is due to different exothermicities of the reaction channels, resulting in different amounts of MMA molecules required for their suppression.

**Figure 9:**
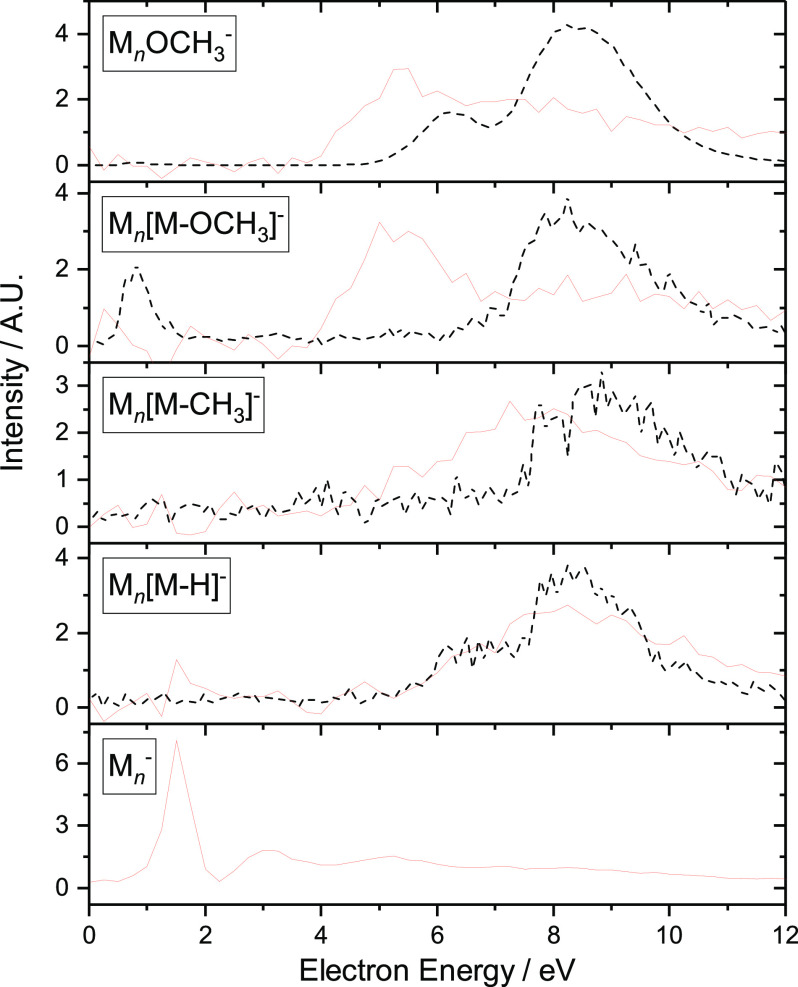
Red: energy-dependent anion yield curves for electron attachment to MMA clusters (sum of all *n*). Black dashed: energy-dependent ion yield curves for isolated MMA (replicated from Figure 6). All curves are area-normalized.

All of the cluster data presented so far were energy-integrated mass spectra. Figure 9 shows the energy-dependent ion yields for selected types of fragment ions. Considerable differences to the corresponding gas-phase ion yields are visible. First of all, the M_*n*_^–^ ions are observed at very low electron energies. It is worth repeating that the rough electron gun in the cluster beam setup has a bad performance at electron energies below 1.5 eV (see the discussion in ref (44)), and therefore, the signal of M_*n*_^–^ ions in Figure 9 most probably corresponds to thermal (near 0 eV) electron attachment. The second clear difference is a new band with a maximum between 5 and 6 eV in the M_*n*_(OCH_3_)^−^ and M_*n*_(M – OCH_3_)^−^ spectra, which was not present in the gas-phase data. The appearance of new bands in the fragmentation spectra of clusters (and in the condensed phase) is a relatively common phenomenon and is typically connected with self-scavenging, a secondary process where in the first step the incident electron electronically excites one cluster constituent, which then possibly undergoes neutral fragmentation, and the slowed-down electron reacts further (see examples in refs (66) and (67)).

Indeed, Schafer et al.^33^ identified the ^1^(n, π*) excited state of MMA at 5.18 eV. Considering the fact that cofragment ions M_*n*_(OCH_3_)^−^ and M_*n*_(M – OCH_3_)^−^, are both formed in same resonance, the most plausible explanation is that the ^1^(n, π*) excited state is dissociative, leading to the cleavage of the 6C–8O bond and neutral dissociation products in the gas phase. In clusters, the outgoing electron is scavenged by the cluster, leading to the formation of M_*n*_(OCH_3_)^−^ and M_*n*_(M – OCH_3_)^−^ fragments. Interesting is the fact that neutral dissociation is accessible from an energetically lower-lying state than DEA despite the high electron affinity of OCH_3_. That further emphasizes the need for detailed studies of neutral dissociation upon electron attachment.^68,69^

## Conclusions

We probed low-energy electron collisions with MMA. The 2D electron energy loss spectrum revealed that the MMA monomer unit efficiently traps electrons with incident energies up to 1 eV and slows them down to energies below 0.2 eV. The excess energy is distributed among vibrational degrees of freedom of the molecule. The excitation efficiency curves for individual vibrational modes revealed a number of shape resonances that were assigned using empirical scaling of virtual orbitals of the molecule. These shape resonances play only a minor role in the dissociative electron attachment—the extensive fragmentation is caused mainly by formation of core-excited resonances above 5 eV.

The cluster experiments show that the electron-induced processes significantly change upon elementary aggregation. First, the aggregated MMA molecules efficiently attach slow electrons, and the intact M_*n*_^–^ is the strongest progression in the mass spectrum. Even though the fragmentation is suppressed in comparison to the isolated molecule, [M – CH_3_]^−^, OCH_3_^–^, and [M – OCH_3_]^−^ channels remain open even for the largest clusters observed in the present experiment with around 10 monomer units. Energy-dependent ion yields for these fragments then indicate that they are formed in a much wider energy range of the impact electrons, possibly due to neutral dissociation and intracluster scavenging processes.

The present results elucidate how electron energy loss and electron-induced bond breaking differ between the isolated MMA molecule and its non-covalently bound clusters. The PMMA resist used in lithography has of course a very different structure, with the MMA units covalently bound via the 3C methyl groups. Nonetheless, it is reasonable to assume that the main effects of the aggregation will be the same, namely, efficient vibrational heating (equivalent to the presently observed IVR), trapping of the slow electrons (equivalent to the present M_*n*_^–^ formation), neutral dissociation, and intramolecular chemistry, including self-scavenging. These effects should be taken into account in modeling the electron propagation through PMMA, e.g., in the secondary electron blur formation.

## Supporting Information Available

Details of the mass spectra presented in Figure 7 (PDF)
