# Supplementary material for: Electron Energy Loss Processes in Methyl Methacrylate: Excitation and Bond Breaking
Source: J Phys Chem A. 2023 Mar 17;127(12):2731–41. doi: 10.1021/acs.jpca.2c09077 (PMC10068740; doi:10.1021/acs.jpca.2c09077)
Supplement: Supplementary file 1 — jp2c09077_si_001.pdf [file jp2c09077_si_001.pdf]

Supporting Information: Electron Energy Loss Processes in Methyl Methacrylate: Excitation and Bond Breaking

Thomas F.M. Luxford, Juraj Fedor,* and Jaroslav Kočišek*

J. Heyrovský Institute of Physical Chemistry of CAS, Dolejškova 3, 18223 Prague, Czech Republic.

E-mail: juraj.fedor@jh-inst.cas.cz; jaroslav.kocisek@jh-inst.cas.cz

Details of Mass Spectra Presented in Fig. 7, Positive Ion Mass Spectrum. page S3
Details of Mass Spectra Presented in Fig. 7, Negative Ion Mass Spectrum. page S4

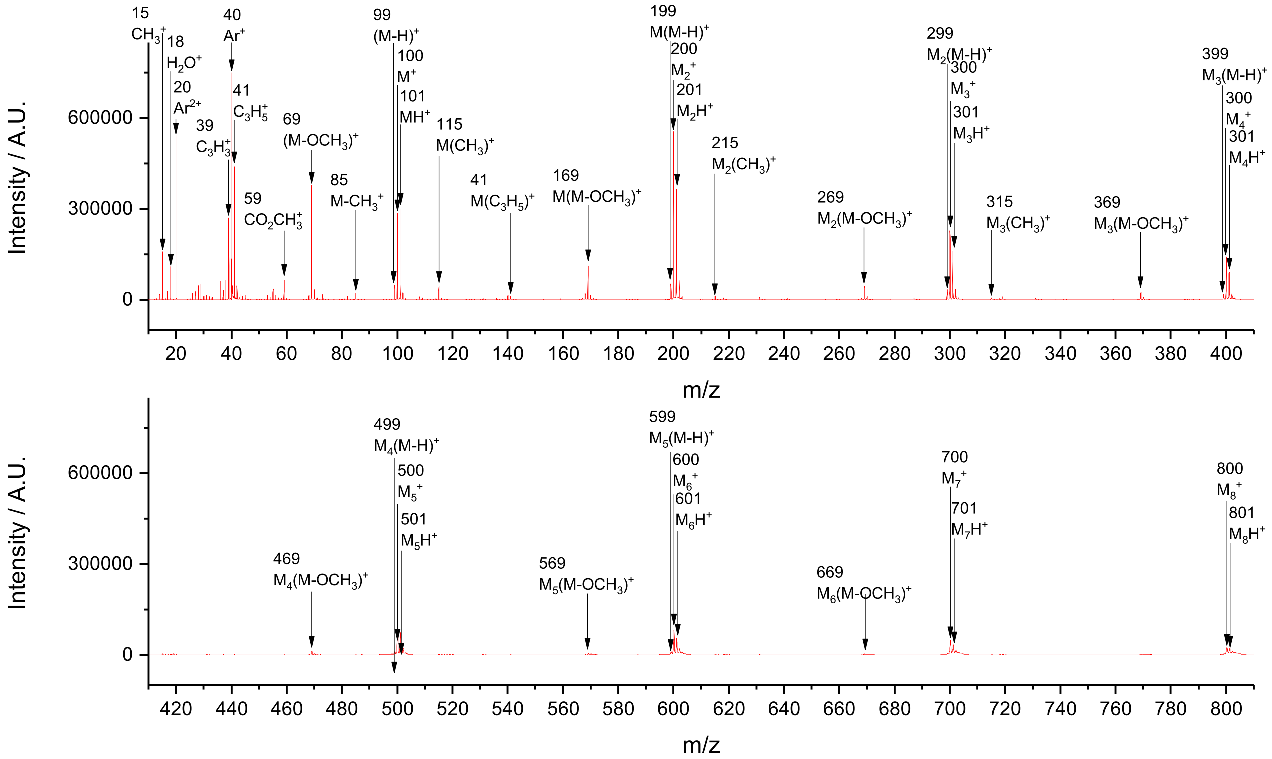

Figure S1: Details of Mass Spectra Presented in Fig. 7, Positive Ion Mass Spectrum. Mass spectrum of positive ions in 10-410 Da range (top) and 410-810 Da range (bottom) recorded at an electron energy of 70 eV.

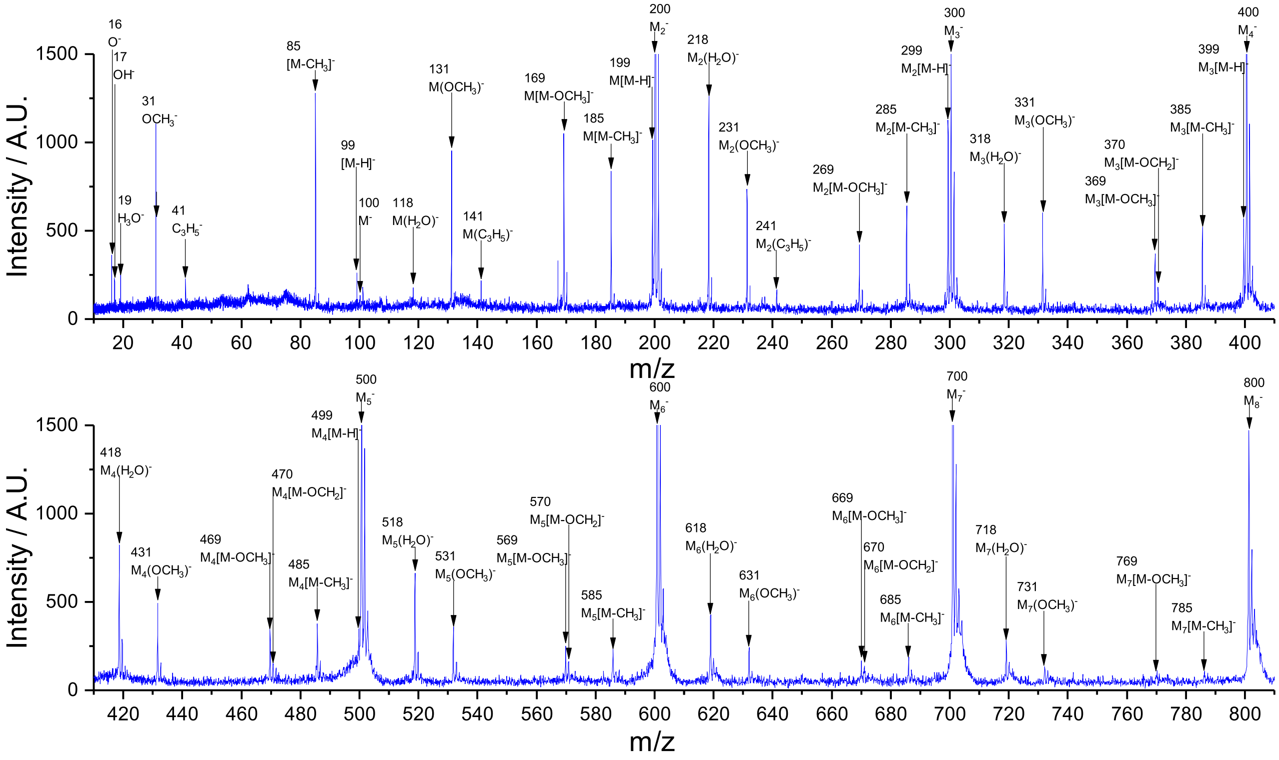

Figure S2: Details of Mass Spectra Presented in Fig. 7, Negative Ion Mass Spectrum. Mass spectrum of negative ions in 10-410 Da range (top) and 410-810 Da range (bottom) created as a sum of individual spectra taken at electron energies from 0 to 15eV.
